# Impact of Bariatric Surgery-Induced Weight Loss on Anterior Eye Health in Patients with Obesity

**DOI:** 10.3390/nu14122462

**Published:** 2022-06-14

**Authors:** Said Karimzad, Paramdeep S. Bilkhu, James S. Wolffsohn, Srikanth Bellary, Hala Shokr, Rishi Singhal, Doina Gherghel

**Affiliations:** 1 Optometry and Vision Sciences Research Group, Aston University, Birmingham B4 7ET, UK; saidekarimzad@hotmail.com (S.K.); p.bilkhu@aston.ac.uk (P.S.B.); hshokr@aston.ac.uk (H.S.); 2 Aston Research Centre for Healthy Ageing (ARCHA), Aston University, Birmingham B4 7ET, UK; s.bellary@aston.ac.uk (S.B.); rishi.singhal2@uhb.nhs.uk (R.S.)

**Keywords:** tear film, obesity, bariatric surgery, ocular surface, lipid, cholesterol

## Abstract

The aim of the present research was to assess the effect of bariatric surgery-induced weight loss on the tear film and ocular surface of patients with obesity. A total of 29 participants with obesity (aged 47.2 ± 10.1 years, 8 male) were measured at baseline and followed up one year after Roux-en-Y gastric bypass (RYGB) surgery. General anthropometric data, as well as serum lipid markers of cholesterol, were assessed in all individuals. Bilateral anterior eye measurements of tear meniscus height (TMH), non-invasive tear breakup time, bulbar and limbal redness and infrared meibography were captured using the Keratograph K5M (Oculus) and ocular surface damage was evaluated using fluorescein sodium and lissamine green staining. Bariatric surgery resulted in significant loss of weight (body mass index *p* < 0.001) and an improvement in the blood lipid profile (*p* < 0.01) in all participants. However, there were no statistically significant differences between the baseline and one-year follow-up for any of the measured clinical ocular surface and tear film variables (all *p* > 0.05). Although there were trends for a reduced TMH and a decrease in meibomian gland dropout after bariatric surgery, these differences were also insignificant (*p* > 0.05). In conclusion, weight reduction through bariatric surgery did not have an effect on the tear film or ocular surface in unselected patients with obesity.

## 1. Introduction

The World Health Organization (WHO) defines obesity as a body mass index (BMI) greater than 30 kg/m^2^ and the international obesity task force has estimated that worldwide there are around 340 million people who meet this criterion, and counting [1].

The impact of obesity on health and its deleterious effects on the body are well known [2]. Overweight and obesity are established risk factors for the development of cardiovascular (CVD) [1] and metabolic diseases such as type 2 diabetes (T2DM) [3]. Obesity is also an important risk factor for other non-communicable diseases such as stroke, asthma and cancer [3].

In addition to its systemic complications, some studies have also established associations between obesity and various ocular pathologies, such as glaucoma [4,5], cataracts [6,7], diabetic retinopathy (DR) [8,9] and floppy eyelid syndrome (FES) [10,11]. Nevertheless, the effect of obesity on the ocular surface’s health is less clear, with many conflicted reports. Indeed, although obesity is associated with abnormal lipid metabolism such as dyslipidaemia [12] which, in turn, is associated with dry eye disease (DED) [13,14], to date there is no research strongly linking obesity to DED. In addition, although high levels of body fat have been associated with dry eye symptoms in a general population, a high body mass index (BMI), which represents the main parameter considered when a person is classified as obese, was not found to be linked to changes in the ocular surface [15]. Moreover, although tear hyperosmolarity, as well as reduced tear stability and volume, was reported in patients suffering from metabolic syndrome (a combination of DM, high blood pressure (BP) and obesity) [16], a systematic review of this disease concluded that all its components were risk factors for dry eye disease with the exception of obesity [17]. 

So, the link between obesity and changes in the ocular surface is controversial, perhaps due to the many confounding environmental and comorbidity factors. In addition, the effect of significant weight loss through either non-surgical or surgical methods on the quality of the ocular surface is also unclear. While an improvement in the circulatory levels of lipids associated with significant weight loss could lead, in theory, to beneficial effects on the quality of the ocular surface, to date there is no research to clearly reflect this. Moreover, although bariatric surgery is associated with great overall beneficial effects, including higher remission of chronic diseases such as DM [18], it can also result in severe nutritional deficits due to restricted diets and changes in the anatomy and physiology of the digestive tract [19] and these deficiencies could have effects at multiple levels, including the ocular surface. Indeed, it has been shown that patients with a history of bariatric surgery demonstrate abnormal tear film and have a higher risk of developing dry eye [20]. Nevertheless, another study, that performed pre- and post-bariatric surgery measurements in different groups of patients, did not find such an effect [21]. Due to the fact that there is no clarity of the effect of weight-induced surgery on the quality of the ocular surface, the aim of this research was to prospectively assess the direct effect of weight loss (from bariatric surgery) on the tear film and ocular surface in unselected patients with obesity that underwent Roux-en-Y gastric bypass (RYGB) surgery.

## 2. Materials and Methods

The study was designed and conducted in accordance with the Declaration of Helsinki and the protocol received positive opinions and governance approval from the hospital and university research ethics committees prior to the commencement of the study.

Participants were recruited from the hospital trusts’ weight management clinics by a specialist consultant following written informed consent. To be suitable candidates for bariatric surgery, the patients had to be between 18 and 65 years old, have a BMI > 40 kg/m^2^ and be compliant with a dietary plan for at least 12 months. Participants were excluded if they had a positive diagnosis of cardio- or cerebrovascular disease and renal disease. All participants were screened for ocular disease and were excluded from the study if they had any ocular pathologies, an intraocular pressure (IOP) > 24 mmHg consistently, moderate to high cataracts and if they had a history of intraocular surgery. The existence of ocular surface damage was neither an inclusion nor exclusion criteria for participants; however, patients with symptomatic red eye (>2 grade using an Efron grading scale) [22,23] were not included in the study.

All included participants underwent RYGB surgery according to a standard technique. Baseline measurements were performed 1 month prior to the participant undergoing surgery and the follow-up was performed 12 months after the procedure. The assessments are detailed below. 

Standard anthropometric measures of height and weight were recorded to determine body mass index (BMI = weight/height). Systolic blood pressure (SBP), diastolic blood pressure (DBP) and heart rate (HR) were measured using an automatic blood pressure monitor (UA-767; A&D Instruments Ltd., Abingdon, UK) to determine mean arterial pressure (MAP = 2/3 DBP + 1/3 SBP) [24]. Intra-ocular pressure (IOP) readings were obtained using non-contact tonometry (Pulsair; Keeler Ltd., Winsor, UK). In addition, blood and plasma samples drawn from the antecubital fossa vein were assessed immediately for TG, total cholesterol (T-CHOL) and HDL-C using the Reflotron Desktop Analyzer (Roche Diagnostics, Burges Hill, UK). Low-density lipoprotein cholesterol (LDL-C) values were calculated as per the Friedewald equation [25].

**Figure 1:**
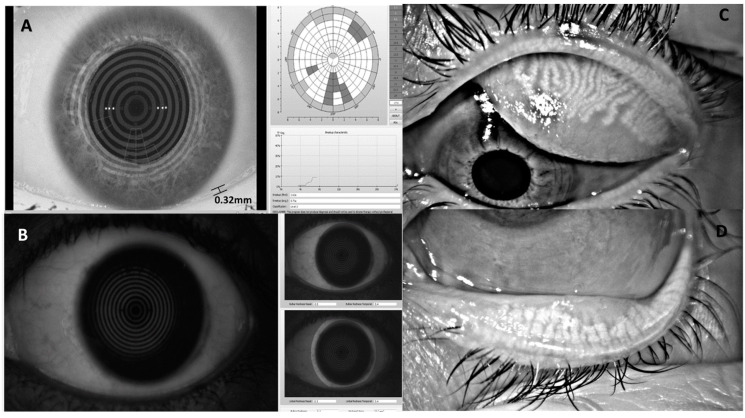
Tear film and ocular surface parameters assessed: (**A**) tear meniscus height (TMH) measured using digital calipers and non-invasive tear breakup time (NIBUT) determined by the objectively assessed disruption of projected infrared light Placido disc reflected from the tear film; (**B**) objectively graded redness of the bulbar (over the white of the eye) and limbal (around the colored iris) regions; (**C**) infrared light imaging of the meibomian glands under the upper (**C**) and lower (**D**) eyelid.

Measures of tear film meniscus height (TMH: average of 3 consecutive readings immediately below the pupil while in primary gaze using the built-in free-hand digital calipers) [26], objective non-invasive breakup time (NIBUT): initial and average of 3 consecutive readings while in primary gaze using the Placido disc and objective image analysis software [27] and objective bulbar and limbal hyperemia (graded against the Efron grading scale) [22,23] were obtained using the Oculus Keratoscope K5M(Optikgerate GmbH, Wetzlar, Germany) (Figure 1). 

Meibomian gland dropout (loss) was calculated after image analysis with ImageJ software (V1.49) based on well-established grading systems [28,29,30] following infrared photography of the upper and lower palpebral surfaces with the K5M [31]. Finally, ocular surface staining was assessed using fluorescein sodium (BioFluoro, Gallowstown, Ireland) and lissamine green (GreenGlo, Hub Pharmaceuticals, Plymouth, MI, USA) ophthalmic dyes applied to the outer canthus [32]. The same measurements were obtained at the follow-up conducted 12 months post-surgery, at the same time of day (±1 h) when the initial measurements were performed for each participant. Staining images captured with the Oculus Keratograph K5m excited with blue light and imaged through a yellow cut-off filter [33] were analyzed with ImageJ software (National Institutes of Health, Bethesda, MD, USA) and the staining area outlined manually and compared to the corneal area. 

### Statistical Analysis

Statistical analyses were performed using Statistica^®^ software (StatSoft Inc., Version 13, Tulsa, OK, USA). As the data did not differ from a normal distribution (Shapiro–Wilks test), differences between the clinical investigations at baseline and follow-up were assessed by using a paired *t*-test. Differences between ocular measurements at baseline and follow-up were assessed by *t*-test or analysis of covariance (ANCOVA) where applicable. Multivariate analyses were performed to test the influence of age, clinical parameters and circulating markers on the ocular variables. Statistical significance was defined at *p* < 0.05. The sample size was calculated using G-power software [34]. Power calculations were based on similar studies that used the K5M system for the assessment for dry eye disease and infrared meibography [23,29,35,36]. Therefore, to provide a statistical power of 80% and medium effect size with an alpha level of 0.05, a sample size of *n* = 27 participants was required.

## 3. Results

A total of 40 participants were recruited for study inclusion to allow for dropout and completed all the baseline measurements; 11 participants were lost to follow-up. The remaining 29 participants (aged 47.2 ± 10.1, range 28–60 years, 11 male) attended the follow-up 12 months post-surgery to complete the study and therefore were included in the analysis. 

The general characteristics of the study group at baseline and follow-up, are outlined in Table 1.

Bariatric surgery resulted in a significant mean reduction in BMI (by 10.82 kg/m^2^; ⸗22%; *p* < 0.001), as well as reductions in SBP (by 15.49 mmHg; ⸗11%; *p* < 0.001), DBP (by 5.62 mmHg; ⸗7%; *p* = 0.039) and HR (by 4.93 bpm; ⸗7%; *p* = 0.046). In addition, blood lipid profiles also showed improvements, with statistically significant reductions in CHOL (by 0.37 mmol/L; ⸗8%; *p* = 0.003), LDL-C (by 0.48 mmol/L; ⸗16%; *p* < 0.001) and TG (by 0.31 mmol/L; ⸗21%; *p* = 0.002), and increases in HDL-C (by 0.27 mmol/L; ⸗21%; *p* < 0.001). (all Table 1)

Pre-surgery, seventy-six percent of participants (*n* = 22) had >5% corneal or conjunctival staining and approximately half (45%, *n* = 13) had an unstable tear film (NIBUT < 10 s). However, there was no statistically significant change between the baseline and at 12 months post-surgery for any of the measured ocular surface characteristics (all *p* > 0.05, Table 2). There was a trend for the amelioration of meibomian gland dropout, a reduction in TMH and a reduction in limbus redness nasally; however, these differences were also statistically insignificant (*p* > 0.05, Table 2). 

No significant correlations (*p* < 0.001 to correct for multiple comparisons) were identified between systemic biomarkers and tear film/ocular surface characteristics (Table 3).

## 4. Discussion

By using participants as their own controls, this study has assessed the effect of significant weight loss after bariatric surgery on the health of the ocular surface and tear film. However, despite a significant reduction in weight and an improvement in the circulatory lipid levels, there was no significant effect of bariatric surgery on the ocular surface health in our participants, even in those with pre-existing signs of ocular surface damage. This finding is similar to an earlier report that included pre- and post-operative measurements in different groups of patients [21]. Follow-up was performed 12 months after the procedure, a period of time that is considered appropriate to allow changes in signs and symptoms to occur [37]. 

Our results could partially be explained by the heterogeneity of the participant cohort. Some of the participants already had a relatively good tear secretion pre-surgery, with tear stability and tear volume of the cohort on average at the borderline of normality [32], while others had various degrees of damage. Indeed, although the link between obesity and various ocular surface damage is not clear, the role of adipokines, secreted by the adipose tissue, in causing systemic chronic inflammation is well established [38]. Moreover, studies performed on animals demonstrated a link between adiponectin, a 30-kDa multimeric protein that is mainly secreted by white adipose tissue, and tear secretion in ageing mice [39]. Therefore, the presence of dry eye and/or various degrees of ocular surface inflammation in individuals with obesity is perfectly plausible. Nevertheless, the average meibomian gland loss (dropout) assessed in our obese patients was not at a level that is believed to impact the overall secretion of meibum [40]. 

All participants included in our study underwent a Roux-en Y gastric bypass (RYGB) procedure, which represents a mixture of restrictive and malabsorptive techniques that are key for improving circulatory CHOL (HDL, LDL and Total) and TG. However, this procedure is also known to greatly increases the risk for various nutritional deficiencies, including those for vitamin B12, folate [41] and vitamin A [42], all very important in the ocular surface health [43,44,45]. In addition, deficiency of vitamin B6, B12 and folate can also cause an increase in the level of circulating homocysteine (Hcy). High Hcy levels are linked to a higher risk for CVD, but also for the occurrence of dry eye [46] as well as of other ocular conditions, such as glaucoma and retinal artery/vein occlusions [47,48]. Indeed, there is some evidence showing that Hcy levels are modified after RYGB [49,50]. Although we did not assess the levels of vitamin B, folate or Hcy, we found that the levels of CHOL and TG were indeed improved after the procedure. If this effect counteracted the one linked to high Hcy levels and, on average, contributed to the lack of significant changes at the ocular surface level can only be a hypothesis at this stage.

The level of meibomian gland dropout in our cohort was similar to what would be expected for this age group in the general population [51] and did not change significantly with weight loss. The role of obesity on the occurrence of meibomian dysfunction is unclear, although dyslipidemia has been found to potentially play a role [52]. In addition, beside its positive impact on circulatory lipids, bariatric surgery is also known to improve insulin resistance [53] and it has been demonstrated that androgenic hormones and insulin may be necessary for the growth and normal functioning of the meibomian gland [54]. Therefore, even if a direct link between the meibomian gland and weight loss after bariatric surgery could not be demonstrated in the present study, it could be hypothesized that it is possible to have a certain beneficial effect on the meibomian gland functioning. More research is necessary to conform this hypothesis. 

The main limitations of our study lie in the fact that the participants were consecutive patients with obesity, recruited from a specialist clinic without a pre-study screening for symptoms or signs of ocular surface disease. Circulatory levels of various nutrients were also not assessed. Nevertheless, our sample was a true representation of the heterogeneity of patients with obesity undergoing this procedure and, in this type of population, it can be concluded that, despite significant positive effects on the measured systemic and circulatory parameters, bariatric surgery does not have measurable effects on the ocular surface health of these individuals.

## Figures and Tables

**Table 1:** The general characteristics of the study groups are outlined.

Parameter	Baseline	Follow-Up	*p*-Value
BMI (kg/m^2^)	49.20 (7.69)	38.38 (7.84)	<0.001 *
SBP (mmHg)	144.24 (14.35)	128.75 (13.23)	<0.001 *
DBP (mmHg)	78.96 (11.35)	73.34 (10.84)	0.039 *
MAP (mmHg)	100.72 (10.58)	92.48 (10.94)	<0.001 *
HR (bpm)	74.10 (13.45)	69.17 (9.88)	0.046 *
CHOL (mmol/L)	4.90 (1.21)	4.53 (0.97)	0.003 *
HDL-C (mmol/L)	1.24 (0.35)	1.51 (0.40)	<0.001 *
LDL-C (mmol/L)	2.97 (1.01)	2.49 (0.81)	<0.001 *
TG (mmol/L)	1.44 (0.72)	1.13 (0.50)	0.002 *

General characteristics of the study group, 1 month prior and 12 months post-surgery. Values are given as mean (±1 standard deviation). Parameters measured: body mass index (BMI); systolic blood pressure (SBP); diastolic blood pressure (DBP); mean arterial pressure (MAP); heart rate (HR); total cholesterol (CHOL); high-density lipoprotein cholesterol (HDL-C); low-density lipoprotein cholesterol (LDL-C); triglycerides (TG). *, *p*-values with an asterisk indicate where *p* < 0.05 reached statistical significance.

**Table 2:** Ocular surface parameters before and after bariatric surgery.

Parameter	Baseline	Follow Up	*p*-Value
TMH (mm)	0.30 (0.10)	0.27 (0.08)	0.074
Fluorescein staining (%)	1.76 (2.46)	2.73 (4.53)	0.333
Lissamine staining (%)	12.10 (9.32)	11.03 (4.72)	0.372
*NIBUT*			
First (sec)	10.08 (7.08)	10.29 (6.72)	0.875
Average (sec)	11.92 (6.71)	12.14 (6.53)	0.849
*Hyperemia*			
Bulbar temporal	0.86 (9.36)	0.86 (0.44)	0.952
Bulbar nasal	0.99 (0.47)	0.93 (0.41)	0.451
Limbal temporal	0.57 (0.32)	0.57 (0.39)	0.948
Limbal nasal	0.71 (0.33)	0.62 (0.32)	0.082
*MG Dropout*			
Superior loss (%)	22.12 (11.73)	18.64 (10.88)	0.062
Inferior loss (%)	16.99 (9.94)	14.39 (11.06)	0.181

Values are given as mean (±1 standard deviation). Parameters measured: tear meniscus height (TMH); staining; non-invasive breakup time (NIBUT) hyperemia; and meibomian gland (MG) dropout and staining at baseline and follow-up.

**Table 3:** Associations (Spearman’s rank correlation) between the combined baseline and follow-up.

		BMI	SBP	DBP	MAP	HR	CHOL	HDL-C	LDL-C	TG
	−	0.310	0.201	0.138	0.179	0.116	0.155	−0.120	0.268	−0.108
Staining	Fluorescein	−0.010	−0.075	−0.050	−0.078	0.025	−0.151	−0.164	−0.150	0.083
	Lissamine	0.219	0.046	−0.176	−0.103	0.341	0.011	−10.49	0.041	0.110
NIBUT	First	0.050	0.115	0.048	0.080	0.322	−00.41	−0.114	0.048	−0.069
	Average	0.033	0.131	0.034	0.080	0.348	−00.86	−0.191	0.001	−0.035
Hyperemia	Bulbar Temporal	−0.129	0.103	−0.032	0.036	−0.191	−0.097	−0.063	−0.107	0.121
	Bulbar Nasal	−0.049	0.114	−0.102	−0.010	−0.048	−0.153	−0.037	−0.096	0.016
	Limbal Temporal	−0.115	0.169	0.024	0.119	−0.307	0.012	−0.094	−0.001	0.181
	Limbal Nasal	0.003	0.245	0.037	0.144	−0.126	0.124	0.062	0.137	0.089
MG Dropout	Upper	0.112	0.310	0.150	0.237	0.049	−0.041	−0.051	0.003	−0.026
	Lower	0.204	0.213	−0.001	0.076	−0.123	0.280	−0.031	0.325	0.083

Body mass index (BMI); systolic blood pressure (SBP); diastolic blood pressure (DBP); mean arterial pressure (MAP); heart rate (HR); total cholesterol (CHOL); high-density lipoprotein cholesterol (HDL-C); low-density lipoprotein cholesterol (LDL-C); triglycerides (TG) with: tear meniscus height (TMH); staining; non-invasive breakup time (NIBUT) hyperemia; and meibomian gland (MG) dropout and staining.

## Data Availability

The data presented in this study are available on request from the corresponding author. The data are not publicly available due to ethical considerations.
